# Di-μ-sulfato-κ^4^ *O*:*O*′-bis­[diaqua­(1*H*-imidazo[4,5-*f*][1,10]phenanthroline)manganese(II)] dihydrate

**DOI:** 10.1107/S1600536810031909

**Published:** 2010-08-18

**Authors:** Ming-Xing Yang, Shen Lin, Li-Juan Chen, Xiao-Hua Chen

**Affiliations:** a College of Chemistry and Materials Science, Fujian Normal University, Fuzhou, Fujian 350007, People’s Republic of China; b State Key Laboratory of Structural Chemistry, Fujian Institute of Research on the Structure of Matter, Chinese Academy of Sciences, Fuzhou, Fujian 350002, People’s Republic of China

## Abstract

In the title centrosymmetric dinuclear compound, [Mn_2_(SO_4_)_2_(C_13_H_8_N_4_)_2_(H_2_O)_4_]·2H_2_O, the Mn^II^ atom is octa­hedrally coordinated by two N atoms from a 1*H*-imidazo[4,5-*f*][1,10]phenanthroline (ip) ligand, two O atoms belonging to two bridging sulfate anions and two water O atoms. In the crystal structure, the complex mol­ecules and the uncoodinated water mol­ecules are connected by O—H⋯O, O—H⋯N and N—H⋯O hydrogen bonds into a three-dimensional network. A π–π stacking inter­action between the pyridyl ring of the ip ligand and the benzene ring of the neighboring ligand [centroid–centroid distance = 3.579 (2) Å] is also observed.

## Related literature

For general background to the crystal engineering of functional materials, see: Aoyama (1998 ▶); Bassani *et al.* (2000 ▶); Kahn (2000 ▶); Matsuda *et al.* (2005 ▶); Miller (2000 ▶); Rowsell *et al.* (2004 ▶). For related structures, see: Gong *et al.* (2009 ▶); Wang *et al.* (2008 ▶); Wu *et al.* (1997 ▶); Yang *et al.* (2010 ▶); Yu (2009 ▶); Zeng *et al.* (2009 ▶).
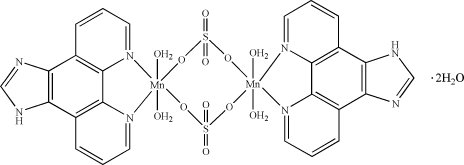


## Experimental

### 

#### Crystal data

[Mn_2_(SO_4_)_2_(C_13_H_8_N_4_)_2_(H_2_O)_4_]·2H_2_O
*M* *_r_* = 850.58
Monoclinic, 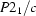

*a* = 10.467 (7) Å
*b* = 9.171 (6) Å
*c* = 17.025 (11) Å
β = 98.758 (12)°
*V* = 1615.2 (18) Å^3^
*Z* = 2
Mo *K*α radiation
μ = 1.00 mm^−1^
*T* = 293 K
1.00 × 0.80 × 0.60 mm

#### Data collection

Rigaku Mercury CCD diffractometer
Absorption correction: multi-scan (*CrystalClear*; Rigaku, 2002 ▶) *T*_min_ = 0.432, *T*_max_ = 1.000
11099 measured reflections
3544 independent reflections
3251 reflections with *I* > 2σ(*I*)
*R*_int_ = 0.024

#### Refinement

*R*[*F* ^2^ > 2σ(*F* ^2^)] = 0.030
*wR*(*F* ^2^) = 0.082
*S* = 1.05
3544 reflections
259 parameters
9 restraints
H atoms treated by a mixture of independent and constrained refinement
Δρ_max_ = 0.30 e Å^−3^
Δρ_min_ = −0.39 e Å^−3^

### 

Data collection: *CrystalClear* (Rigaku, 2002 ▶); cell refinement: *CrystalClear*; data reduction: *CrystalClear*; program(s) used to solve structure: *SHELXS97* (Sheldrick, 2008 ▶); program(s) used to refine structure: *SHELXL97* (Sheldrick, 2008 ▶); molecular graphics: *SHELXTL* (Sheldrick, 2008 ▶) and *DIAMOND* (Brandenburg, 1999 ▶); software used to prepare material for publication: *SHELXTL*.

## Supplementary Material

Crystal structure: contains datablocks global, I. DOI: 10.1107/S1600536810031909/hy2338sup1.cif

Structure factors: contains datablocks I. DOI: 10.1107/S1600536810031909/hy2338Isup2.hkl

Additional supplementary materials:  crystallographic information; 3D view; checkCIF report

## Figures and Tables

**Table 1:** Selected bond lengths (Å)

Mn1—O1	2.1366 (17)
Mn1—O3^i^	2.1641 (16)
Mn1—O5	2.2590 (16)
Mn1—O6	2.1751 (17)
Mn1—N1	2.2718 (19)
Mn1—N2	2.2715 (19)

Symmetry code: (i) 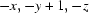.

**Table 2:** Hydrogen-bond geometry (Å, °)

*D*—H⋯*A*	*D*—H	H⋯*A*	*D*⋯*A*	*D*—H⋯*A*
O5—H1⋯O2^i^	0.85 (1)	1.95 (1)	2.789 (2)	165 (3)
O5—H2⋯N4^ii^	0.86 (3)	1.99 (3)	2.824 (2)	164 (3)
O6—H3⋯O7^iii^	0.84 (1)	1.80 (1)	2.644 (3)	172 (3)
O6—H4⋯O2	0.84 (2)	1.97 (1)	2.745 (2)	154 (2)
O7—H5⋯O2^iv^	0.85 (1)	1.98 (1)	2.828 (3)	171 (3)
O7—H6⋯O4	0.84 (1)	2.04 (2)	2.833 (3)	157 (3)
N3—H3*B*⋯O4^v^	0.86	2.04	2.890 (2)	167

Symmetry codes: (i) 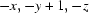; (ii) 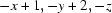; (iii) 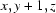; (iv) 
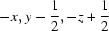
; (v) 
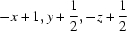
.

**Fig. 1.**
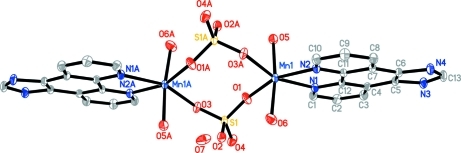
Molecular structure of the title compound. H atoms have been omitted for clarity. [Symmetry code: (A) -x, 1-y, -z.]

**Fig. 2.**
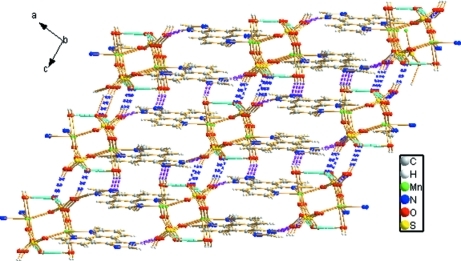
The three-dimensional hydrogen bonding network in the title compound.

## supplementary crystallographic information

### Comment

An important aspect of crystal engineering is to understand and attempt to control the manner in which molecules are arranged in crystal lattices through the use of noncovalent interactions such as electrostatic interactions, hydrogen bonding, dispersion and induction forces, and π-π stacking interactions. These materials have attracted much interest due to their strong potential for a variety of applications including gas storage (Matsuda *et al.*, 2005; Rowsell *et al.*, 2004), catalytic properties (Aoyama, 1998; Bassani *et al.*, 2000) and magnetism (Kahn, 2000; Miller, 2000). One approach to forming networks of discrete transition metal complexes is to use a chelating ligand that has additional interactional functionality attached to its backbone, such as additional coordination sites or hydrogen bonding groups, or extended π systems. 1*H*-Imidazo[5,f][1,10]phenanthroline (ip) has been used to form metal complexes with novel supramolecular architectures due to their excellent coordinating ability, large conjugated systems and strong hydrogen bonding donor and acceptor groups (Gong *et al.*, 2009; Wang *et al.*, 2008; Wu *et al.*, 1997; Yang *et al.*, 2010; Yu, 2009; Zeng *et al.*, 2009). In the present paper,we hydrothermally synthesized a new coordination complex constructed from MnSO_4_ and ip.

The title dimeric complex is generated by an inversion center (Fig. 1). The Mn^II^ atom is six-coordinated by two N atoms from one ip ligand, two O atoms from water molecules and two O atoms from two sulfate anions in a distorted octahedral geometry (Table 1). The equatorial plane is defined by N2, O6, O1 and O5 and the axial coordination sites are occupied by N1 and O3^i^ atoms [symmetry code: (i) -x, 1-y, -z]. The sulfate anion acts as a bidentate bridging ligand connecting two Mn^II^ ions, thus generating a binuclear complex. The hydrogen bonds play a key role in the structural stability (Table 2). The uncoordinated water molecule is a hydrogen bond acceptor from the coordinated water, and a hydrogen bond donor to two O atoms of two sulfate anions in two neighboring complex molecules. So each free water is hydrogen bonded to three different complex molecules. The ip ligand is a hydrogen bond donor through the imidazolyl NH group to a sulfate O atom of an adjacent complex molecule and a hydrogen bond acceptor from the coordinated water molecule (O5) of another adjacent complex molecule through the other imidazolyl N atom, forming a three-dimensional network structure, as illustrated in Fig. 2. There is also a π–π stacking interaction between the pyridyl ring of the ip ligand and the benzene ring of the neighboring ip ligand, with a centroid–centroid distance of 3.579 (2) Å.

### Experimental

The ip ligand was synthesized according to literature (Wu *et al.*, 1997). A mixture of MnSO_4_, ip and H_2_O in a molar ratio of 1:1:556 was stirred for an hour, then sealed in an 18 ml Teflon-lined stainless steel reactor and heated for 3 d at 433 K and autogeneous pressure. After allowing the reaction mixture to cool down to room temperature, yellow crystals were collected, washed with water and dried at room temperature.

### Refinement

C- and N-bound H atoms were positioned geometrically and refined as riding atoms, with C—H = 0.93 and N—H = 0.86 Å and *U*_iso_(H) = 1.2*U*_eq_(C,N). The water H atoms were located in a difference Fourier map and refined isotropically, with restraints of O—H = 0.84 (1) and H···H = 1.44 (1) Å.

### Crystal data

**Table:**

[Mn_2_(SO_4_)_2_(C_13_H_8_N_4_)_2_(H_2_O)_4_]·2H_2_O	*F*(000) = 868
*M**_r_* = 850.58	*D*_x_ = 1.749 Mg m^−^^3^
Monoclinic, *P*2_1_/*c*	Mo *K*α radiation, λ = 0.71073 Å
Hall symbol: -P 2ybc	Cell parameters from 4851 reflections
*a* = 10.467 (7) Å	θ = 3.3–27.4°
*b* = 9.171 (6) Å	µ = 1.00 mm^−^^1^
*c* = 17.025 (11) Å	*T* = 293 K
β = 98.758 (12)°	Prism, yellow
*V* = 1615.2 (18) Å^3^	1.00 × 0.80 × 0.60 mm
*Z* = 2	

### Data collection

**Table:**

Rigaku Mercury CCD diffractometer	3544 independent reflections
Radiation source: fine-focus sealed tube	3251 reflections with *I* > 2σ(*I*)
graphite	*R*_int_ = 0.024
Detector resolution: 14.6306 pixels mm^-1^	θ_max_ = 27.4°, θ_min_ = 2.5°
ω scan	*h* = −13→13
Absorption correction: multi-scan (*CrystalClear*; Rigaku, 2002)	*k* = −11→11
*T*_min_ = 0.432, *T*_max_ = 1.000	*l* = −21→21
11099 measured reflections	

### Refinement

**Table:**

Refinement on *F*^2^	Primary atom site location: structure-invariant direct methods
Least-squares matrix: full	Secondary atom site location: difference Fourier map
*R*[*F*^2^ > 2σ(*F*^2^)] = 0.030	Hydrogen site location: inferred from neighbouring sites
*wR*(*F*^2^) = 0.082	H atoms treated by a mixture of independent and constrained refinement
*S* = 1.05	*w* = 1/[σ^2^(*F*_o_^2^) + (0.0437*P*)^2^ + 0.5623*P*] where *P* = (*F*_o_^2^ + 2*F*_c_^2^)/3
3544 reflections	(Δ/σ)_max_ = 0.001
259 parameters	Δρ_max_ = 0.30 e Å^−^^3^
9 restraints	Δρ_min_ = −0.39 e Å^−^^3^

### Fractional atomic coordinates and isotropic or equivalent isotropic displacement parameters (Å^2^)

**Table:**

	*x*	*y*	*z*	*U*_iso_*/*U*_eq_	
Mn1	0.18299 (2)	0.67998 (3)	0.039544 (16)	0.02452 (10)	
S1	0.03061 (4)	0.40185 (4)	0.11921 (2)	0.02399 (11)	
O1	0.13383 (12)	0.47205 (14)	0.08303 (9)	0.0374 (3)	
O2	−0.06301 (12)	0.51435 (14)	0.13804 (8)	0.0316 (3)	
O3	−0.03679 (12)	0.29274 (14)	0.06387 (8)	0.0311 (3)	
O4	0.08663 (14)	0.32790 (15)	0.19348 (9)	0.0400 (3)	
O5	0.29524 (13)	0.55706 (16)	−0.04252 (8)	0.0339 (3)	
H1	0.2295 (19)	0.519 (3)	−0.0712 (14)	0.074 (9)*	
H2	0.329 (3)	0.626 (3)	−0.0665 (16)	0.096 (12)*	
O6	0.05988 (16)	0.77338 (17)	0.11855 (10)	0.0484 (4)	
H3	0.065 (2)	0.8546 (15)	0.1421 (14)	0.059 (8)*	
H4	0.012 (2)	0.7107 (19)	0.1349 (14)	0.056 (8)*	
O7	0.0949 (3)	0.01928 (19)	0.20000 (11)	0.0714 (6)	
H5	0.090 (3)	0.008 (3)	0.2492 (7)	0.076 (10)*	
H6	0.088 (3)	0.1066 (16)	0.1844 (15)	0.084 (10)*	
N1	0.37913 (14)	0.68351 (15)	0.11788 (9)	0.0257 (3)	
N2	0.25995 (13)	0.91094 (15)	0.03511 (9)	0.0252 (3)	
N3	0.74601 (14)	1.01809 (18)	0.20156 (9)	0.0321 (3)	
H3B	0.8055	0.9678	0.2297	0.039*	
N4	0.64434 (15)	1.21029 (17)	0.13915 (10)	0.0338 (4)	
C1	0.43732 (18)	0.56818 (19)	0.15534 (11)	0.0303 (4)	
H1A	0.3930	0.4800	0.1522	0.036*	
C2	0.56195 (18)	0.5734 (2)	0.19914 (12)	0.0333 (4)	
H2B	0.5993	0.4901	0.2240	0.040*	
C3	0.62820 (17)	0.7031 (2)	0.20480 (12)	0.0314 (4)	
H3C	0.7105	0.7093	0.2343	0.038*	
C4	0.56986 (15)	0.82664 (18)	0.16534 (10)	0.0239 (3)	
C5	0.62700 (15)	0.96855 (19)	0.16508 (10)	0.0257 (4)	
C6	0.56533 (16)	1.08776 (18)	0.12661 (10)	0.0253 (3)	
C7	0.43740 (15)	1.07578 (18)	0.08161 (10)	0.0236 (3)	
C8	0.36759 (18)	1.19078 (18)	0.04063 (12)	0.0302 (4)	
H8A	0.4032	1.2838	0.0414	0.036*	
C9	0.24595 (18)	1.1648 (2)	−0.00075 (12)	0.0337 (4)	
H9A	0.1977	1.2403	−0.0269	0.040*	
C10	0.19618 (16)	1.0231 (2)	−0.00289 (11)	0.0306 (4)	
H10A	0.1150	1.0060	−0.0321	0.037*	
C11	0.37862 (15)	0.93635 (17)	0.07832 (10)	0.0220 (3)	
C12	0.44406 (15)	0.81270 (17)	0.12160 (10)	0.0222 (3)	
C13	0.75016 (19)	1.1614 (2)	0.18408 (12)	0.0370 (4)	
H13A	0.8209	1.2205	0.2020	0.044*	

### Atomic displacement parameters (Å^2^)

**Table:**

	*U*^11^	*U*^22^	*U*^33^	*U*^12^	*U*^13^	*U*^23^
Mn1	0.02168 (14)	0.02247 (15)	0.02823 (17)	−0.00592 (9)	−0.00001 (11)	−0.00163 (10)
S1	0.0223 (2)	0.0203 (2)	0.0272 (2)	−0.00508 (14)	−0.00289 (16)	0.00196 (15)
O1	0.0317 (7)	0.0248 (6)	0.0578 (9)	−0.0043 (5)	0.0138 (6)	0.0050 (6)
O2	0.0289 (6)	0.0270 (6)	0.0384 (7)	−0.0024 (5)	0.0037 (5)	−0.0046 (5)
O3	0.0290 (6)	0.0253 (6)	0.0354 (8)	−0.0023 (5)	−0.0067 (5)	−0.0044 (5)
O4	0.0475 (8)	0.0336 (7)	0.0330 (8)	−0.0034 (6)	−0.0129 (6)	0.0068 (6)
O5	0.0305 (7)	0.0349 (7)	0.0361 (8)	−0.0022 (5)	0.0040 (6)	−0.0011 (6)
O6	0.0599 (9)	0.0302 (8)	0.0626 (10)	−0.0153 (7)	0.0334 (8)	−0.0142 (7)
O7	0.1428 (19)	0.0306 (9)	0.0456 (11)	−0.0169 (10)	0.0295 (12)	−0.0072 (7)
N1	0.0253 (7)	0.0215 (7)	0.0292 (8)	−0.0046 (5)	0.0008 (6)	0.0003 (5)
N2	0.0209 (6)	0.0247 (7)	0.0287 (8)	−0.0030 (5)	0.0000 (6)	0.0014 (6)
N3	0.0235 (7)	0.0369 (9)	0.0323 (9)	−0.0062 (6)	−0.0073 (6)	0.0023 (7)
N4	0.0326 (8)	0.0295 (8)	0.0376 (9)	−0.0113 (6)	−0.0004 (7)	0.0004 (7)
C1	0.0348 (9)	0.0221 (8)	0.0330 (10)	−0.0035 (7)	0.0026 (8)	0.0010 (7)
C2	0.0363 (9)	0.0266 (9)	0.0352 (11)	0.0049 (7)	0.0002 (8)	0.0060 (7)
C3	0.0244 (8)	0.0342 (10)	0.0335 (10)	0.0018 (7)	−0.0023 (7)	0.0032 (8)
C4	0.0217 (7)	0.0251 (8)	0.0240 (9)	−0.0023 (6)	0.0006 (6)	−0.0010 (6)
C5	0.0200 (7)	0.0293 (9)	0.0263 (9)	−0.0046 (6)	−0.0011 (6)	−0.0012 (7)
C6	0.0253 (8)	0.0234 (8)	0.0266 (9)	−0.0068 (6)	0.0022 (7)	−0.0018 (6)
C7	0.0241 (8)	0.0224 (8)	0.0241 (8)	−0.0032 (6)	0.0032 (7)	−0.0012 (6)
C8	0.0327 (9)	0.0207 (8)	0.0364 (11)	−0.0025 (6)	0.0029 (8)	0.0005 (7)
C9	0.0319 (9)	0.0278 (9)	0.0393 (11)	0.0050 (7)	−0.0019 (8)	0.0064 (8)
C10	0.0218 (8)	0.0316 (9)	0.0362 (10)	0.0006 (7)	−0.0023 (7)	0.0042 (8)
C11	0.0196 (7)	0.0220 (8)	0.0240 (9)	−0.0025 (6)	0.0024 (6)	−0.0007 (6)
C12	0.0214 (7)	0.0216 (8)	0.0232 (9)	−0.0030 (6)	0.0019 (6)	−0.0008 (6)
C13	0.0318 (9)	0.0387 (11)	0.0380 (11)	−0.0177 (8)	−0.0029 (8)	−0.0020 (8)

### Geometric parameters (Å, °)

**Table:**

Mn1—O1	2.1366 (17)	N3—H3B	0.8600
Mn1—O3^i^	2.1641 (16)	N4—C13	1.325 (3)
Mn1—O5	2.2590 (16)	N4—C6	1.392 (2)
Mn1—O6	2.1751 (17)	C1—C2	1.401 (3)
Mn1—N1	2.2718 (19)	C1—H1A	0.9300
Mn1—N2	2.2715 (19)	C2—C3	1.373 (3)
S1—O1	1.4711 (14)	C2—H2B	0.9300
S1—O4	1.4753 (16)	C3—C4	1.408 (2)
S1—O3	1.4779 (14)	C3—H3C	0.9300
S1—O2	1.4910 (14)	C4—C12	1.416 (2)
O3—Mn1^i^	2.1641 (16)	C4—C5	1.433 (2)
O5—H1	0.85 (1)	C5—C6	1.383 (2)
O5—H2	0.86 (3)	C6—C7	1.442 (2)
O6—H3	0.84 (1)	C7—C8	1.406 (2)
O6—H4	0.84 (2)	C7—C11	1.416 (2)
O7—H5	0.85 (1)	C8—C9	1.379 (3)
O7—H6	0.84 (1)	C8—H8A	0.9300
N1—C1	1.333 (2)	C9—C10	1.398 (3)
N1—C12	1.363 (2)	C9—H9A	0.9300
N2—C10	1.338 (2)	C10—H10A	0.9300
N2—C11	1.364 (2)	C11—C12	1.464 (2)
N3—C13	1.350 (3)	C13—H13A	0.9300
N3—C5	1.381 (2)		
			
O1—Mn1—O3^i^	101.97 (6)	N1—C1—C2	123.22 (16)
O1—Mn1—O6	86.60 (7)	N1—C1—H1A	118.4
O3^i^—Mn1—O6	92.60 (8)	C2—C1—H1A	118.4
O1—Mn1—O5	86.81 (6)	C3—C2—C1	119.08 (17)
O3^i^—Mn1—O5	85.67 (7)	C3—C2—H2B	120.5
O6—Mn1—O5	172.68 (5)	C1—C2—H2B	120.5
O1—Mn1—N2	161.64 (6)	C2—C3—C4	119.07 (17)
O3^i^—Mn1—N2	94.33 (5)	C2—C3—H3C	120.5
O6—Mn1—N2	84.25 (6)	C4—C3—H3C	120.5
O5—Mn1—N2	102.96 (6)	C3—C4—C12	118.63 (15)
O1—Mn1—N1	93.04 (6)	C3—C4—C5	125.55 (16)
O3^i^—Mn1—N1	160.02 (5)	C12—C4—C5	115.82 (15)
O6—Mn1—N1	101.47 (8)	N3—C5—C6	106.07 (15)
O5—Mn1—N1	82.04 (7)	N3—C5—C4	130.22 (16)
N2—Mn1—N1	73.31 (5)	C6—C5—C4	123.70 (15)
O1—S1—O4	109.79 (9)	C5—C6—N4	110.00 (16)
O1—S1—O3	109.82 (9)	C5—C6—C7	121.34 (15)
O4—S1—O3	108.90 (9)	N4—C6—C7	128.66 (16)
O1—S1—O2	109.62 (9)	C8—C7—C11	117.88 (16)
O4—S1—O2	108.80 (9)	C8—C7—C6	125.22 (15)
O3—S1—O2	109.88 (8)	C11—C7—C6	116.90 (15)
S1—O1—Mn1	140.06 (8)	C9—C8—C7	119.55 (16)
S1—O3—Mn1^i^	130.55 (8)	C9—C8—H8A	120.2
Mn1—O5—H1	96 (2)	C7—C8—H8A	120.2
Mn1—O5—H2	102 (2)	C8—C9—C10	119.05 (16)
H1—O5—H2	112.2 (16)	C8—C9—H9A	120.5
Mn1—O6—H3	129.9 (16)	C10—C9—H9A	120.5
Mn1—O6—H4	112.2 (16)	N2—C10—C9	123.04 (17)
H3—O6—H4	116.3 (16)	N2—C10—H10A	118.5
H5—O7—H6	114.1 (16)	C9—C10—H10A	118.5
C1—N1—C12	118.64 (15)	N2—C11—C7	122.01 (15)
C1—N1—Mn1	125.14 (11)	N2—C11—C12	117.19 (14)
C12—N1—Mn1	116.06 (11)	C7—C11—C12	120.81 (15)
C10—N2—C11	118.41 (15)	N1—C12—C4	121.34 (15)
C10—N2—Mn1	125.42 (12)	N1—C12—C11	117.28 (15)
C11—N2—Mn1	116.10 (11)	C4—C12—C11	121.39 (14)
C13—N3—C5	106.16 (15)	N4—C13—N3	113.86 (16)
C13—N3—H3B	126.9	N4—C13—H13A	123.1
C5—N3—H3B	126.9	N3—C13—H13A	123.1
C13—N4—C6	103.91 (16)		

Symmetry codes: (i) −*x*, −*y*+1, −*z*.

### Hydrogen-bond geometry (Å, °)

**Table:**

*D*—H···*A*	*D*—H	H···*A*	*D*···*A*	*D*—H···*A*
O5—H1···O2^i^	0.85 (1)	1.95 (1)	2.789 (2)	165 (3)
O5—H2···N4^ii^	0.86 (3)	1.99 (3)	2.824 (2)	164 (3)
O6—H3···O7^iii^	0.84 (1)	1.80 (1)	2.644 (3)	172 (3)
O6—H4···O2	0.84 (2)	1.97 (1)	2.745 (2)	154 (2)
O7—H5···O2^iv^	0.85 (1)	1.98 (1)	2.828 (3)	171 (3)
O7—H6···O4	0.84 (1)	2.04 (2)	2.833 (3)	157 (3)
N3—H3B···O4^v^	0.86	2.04	2.890 (2)	167

Symmetry codes: (i) −*x*, −*y*+1, −*z*; (ii) −*x*+1, −*y*+2, −*z*; (iii) *x*, *y*+1, *z*; (iv) −*x*, *y*−1/2, −*z*+1/2; (v) −*x*+1, *y*+1/2, −*z*+1/2.
